# Part II: understanding pain in pigs—pain assessment in pigs with spontaneously occurring diseases or injuries

**DOI:** 10.1186/s40813-025-00420-1

**Published:** 2025-03-12

**Authors:** Julia Kschonek, Kathrin Deters, Moana Miller, Jennifer Reinmold, Lara Twele, Ilka Emmerich, Sabine Kästner, Nicole Kemper, Lothar Kreienbrock, Isabel Hennig-Pauka, Michael Wendt, Elisabeth grosse Beilage

**Affiliations:** 1 Institute for Biometry, Epidemiology and Information Processing (IBEI), University of Veterinary Medicine, Foundation, Bünteweg 2, 30559 Hannover, Germany; 2 Field Station for Epidemiology, University of Veterinary Medicine, Foundation, Büscheler Str. 9, 49456 Hannover, Bakum, Germany; 3 Institute for Animal Hygiene, Animal Welfare and Farm Animal Behavior, University of Veterinary Medicine, Foundation, Bischofsholer Damm 15, 30173 Hannover, Germany; 4 Clinic for Horses, University of Veterinary Medicine, Foundation, Bünteweg 9, 30559 Hannover, Germany; 5 Institute of Pharmacology, Pharmacy and Toxicology, Faculty of Veterinary Medicine, University Leipzig, An Den Tierkliniken 39, 04103 Leipzig, Germany; 6 Clinic for Small Animals, University of Veterinary Medicine, Foundation, Bünteweg 2, 30559 Hannover, Germany; 7 Clinic for Swine and Small Ruminants, Forensic Medicine and Ambulatory Service, University of Veterinary Medicine, Foundation, Bischofsholer Damm 15, 30173 Hannover, Germany

**Keywords:** Pigs, Pain, Assessment, Spontaneously occurring diseases and injuries

## Abstract

**Background:**

Pain in pigs needs to be managed and treated to the benefit of individual pigs. It is imperative for veterinarians and farmers to assure that pigs do not suffer from unnecessary pain that can be relieved. This review focusses on pain related to spontaneously occurring diseases and injuries since this topic is often neglected. The aim is to identify ways to accelerate knowledge and evidence in this area to prevent painful conditions in pigs in the future.

**Methods:**

A scoping review was conducted with results from a search of the electronic databases VetSearch and CABI Rxiv. The findings of selected publications are narratively synthesized and reported orienting on the PRISMA ScR guideline.

**Results:**

The results emphasize that pigs experience pain due to spontaneously occurring diseases and injuries, but systematic knowledge about this topic is scarce. More research is especially needed for rare diseases (such as UTIs). Moreover, research conducted about the topic pain in pigs should involve standardized protocols to document, analyse and share results on pain detection beyond a projects’ timeframe. The findings of this review suggest that such a protocol would comprise validated pain identification measures over time and in relation to administered pain treatment.

**Conclusion:**

The results of this study invite veterinary practitioners to reconsider in each pig patient whether pain and related indicators are present, how to handle the situation and document the process to ensure the welfare of individual compromised pigs.

**Supplementary Information:**

The online version contains supplementary material available at 10.1186/s40813-025-00420-1.

## Introduction

Pain in pigs can be induced by spontaneously occurring diseases and injuries as well as by damaging management procedures. In the literature, the focus to elaborate on signs of pain, pain mechanisms and therapy of pigs is often put on the latter topic and neglects common diseases such as pain due to gastric ulcer, claw avulsion or respiratory disorders. Irrespective of the reason for pain sensitation in pigs, pain-related conditions need to be carefully examined and treated by veterinarians and farmers. For this means, thorough knowledge is needed to examine, assess and therapize related conditions properly. In a previous article, - part I - understanding pain in pigs [1], a narrative synthesis of common and latest published literature was generated for gathering basic knowledge in this respect. The former manuscript also provides an overview of dosages and administration of analgesic agents. This subsequent article - part II - focusses more specifically on pain induced by spontaneously occurring diseases and injuries. This scoping review summarizes how pain in specific diseases is addressed in theory and in veterinary practice. Not anticipating the discussion of results, it will be shown that even for common diseases like lameness in pigs, descriptions of the assessment and alleviation of pain are often indirect or vague. Hence, this review will help readers to learn about the current state of pain relief approaches and findings, and thus what is needed to accelerate evidence about pain in spontaneously occurring diseases and injuries in the future.

## Method

The review was conducted in accordance with the PRISMA-ScR guideline [2] which is dedicated to organize the report of scoping reviews. In contrast to systematic reviews that answer one particular question with the help of specific results and evidence, a scoping review aims to answer generic questions and to provide an initial and structured overview of findings in the field [2, 3]. The research question to be answered in this study is: what is known about the identification and evaluation of pain in pigs with spontaneously occurring diseases or injuries? For this review, pain is defined as “[a]n unpleasant sensory and emotional experience associated with, or resembling that associated with, actual or potential tissue damage […]” ([4], Text Box 2). Depending on its neuroanatomical origin, pain can result from the activation of nociceptors (nociceptive pain), which are among the cardinal symptoms of inflammation (inflammatory pain), lesions of neural tissue (neuropathic pain) and combinations of these conditions [1]. Following this definition, a review protocol was predefined and discussed by the author group (experts in porcine health management, pain, anaesthesia, pharmacology and research methodology in veterinary medicine). The discussion concerned the quality and comprehensibility of the eligibility criteria for the selected papers, the search mode, the database and the review documentation.

For the search of latest publications, the databases VetSearch and CABI search Rxiv were used to identify papers matching the terms for *animals* (pigs), the *focus* of this article (pain) and terms for specific diseases or injuries (*topics*). Many terms were defined, and specifications were established during the search. To enable readers to follow the process for each topic of the manuscript, the metrics used herein are outlined in the supplementary material (Additional file S1).

Papers were included if accessible and peer reviewed or books (chapters) published between 2015 and end of March 2023 besides standard literature on pig diseases and work found through snowballing technique. Included languages are English and German. Concerning the topic, papers are included if they elaborate on the search terms in more than one sentence (buzz-words), i.e. contribute to the topic with descriptive or detailed insight.

## Pain induced by spontaneously occurring diseases or injuries in pigs

### Locomotor diseases

Locomotor diseases include a wide range of infectious, non-infectious or degenerative diseases and injuries, including purulent or non-purulent arthritis, osteoarthritis, osteochondrosis, fractures, tenosynovitis, contusions, muscle tearing, dewclaw injuries or fractures, and coronary band and other foot lesions [5, 6]. Abnormalities in the locomotor system often lead to lameness, which manifests as reduced weight bearing on at least one leg, an inability to stand up in the hindquarters (hind leg weakness) or sitting or lying posture. Lame animals exhibit an abnormal gait, characterised by asymmetrical weight distribution, steep walking, increased stride frequency, shortened stride lengths and/or an arching of the spine. Some animals also exhibit rapid changes between loading and unloading of a limb (tapping) [7, 8].

Lameness in pigs often manifests as changes in behaviour. Lame pigs reduce their activity and exploratory behaviour and reduce their interactions with pen mates. It has also been observed that lame sows lie down on walls more often than their non-lame counterparts [9–11]. Sows with artificially induced arthritis showed longer periods of lying down and shorter periods of standing up [11–15]. Overgrowth of claws in sows leads to shorter stance phases and feed intake times [16]. Due to lameness and associated pain, behaviour changes; affected pigs eat and drink less or not at all, as their condition does not allow them to compete with non-lame pigs for feed and water [27]. Lame sows consume significantly less water than healthy sows [17]. This increases the risk of lame animals not being able to fulfil their needs sufficiently and suffer from hunger and thirst [10, 18]. Therefore, lame sows should be housed in hospital pens where they can recover and not have to compete with healthy sows for water and feed. A feed reward study revealed that moderately to severely lame sows received fewer rewards than mildly lame and non-lame sows [18].

Lameness and associated expenses have a significant economic impact. Lame animals require additional labour, from identification to treatment, which implies higher veterinary costs. Lameness in sows can also have a negative impact on reproductive performance [12, 19]. On average, sows that leave the herd due to lameness are younger than those that are removed for other reasons [20]. Lame sows are less efficient at breeding and are more likely to be culled due to lameness [10, 21]. Musculoskeletal problems are a leading cause of culling in pig herds [22, 23].

Lameness serves as a key indicator for animal welfare assessment in pig farming [24]. Lameness is usually easy to recognize and is a clear sign of pain [12, 24, 25]. The observed behavioural changes in movement disorders are most likely caused by limited mobility, pain or discomfort and sickness behaviour [10]. Lameness is rated as extremely painful by farmers and veterinarians, especially when there is minimal weight bearing [26]. In the same survey, farmers stated that recognizing pain in pigs was difficult and that they were unsure how to address it. However, the assessment of pain is often subjective and, therefore, difficult to quantify [10]. Lameness can cause both pain and stress. Stress is associated with changes in the immune system and increases the susceptibility of animals to other diseases. If lameness persists, additional diseases can develop and affect animal health beyond lameness [10]. In fattening pigs, fractures, osteochondrosis dissecans and infectious arthritis are considered particularly painful [23].

In addition to the visual observation of animals, technology such as pressure mats, force plates, motion capture systems and accelerometers can be used to record animal movement and limb loading [14, 15, 27]. The evaluation of these electronic systems enables the identification of lameness [6]. Using an accelerometer, previous studies showed that lame sows take more steps per minute than healthy animals [14]. Measurements with a force plate showed that lame sows have a lower contralateral hind leg load. Pressure mat analyses showed that lame animals have an asymmetrical gait, probably due to the relief of the affected limb to reduce pain [11, 28]. One advantage of electronic detection systems is their objectivity. Visual observations are subjective and have high variability in reproducibility depending on the training and experience of the observer. However, visual observations are inexpensive and easy to integrate into daily work routines [29].

Foot and joint problems were cited by farmers as the most common reason for administering NSAIDs [30]. In countries where opioid analgesics are licenced, buprenorphine can be used to reduce gait asymmetry, while the NSAID meloxicam can be used to reduce stride frequency, thus leading to better symmetry of hind leg movement in pigs with spontaneously occurring lameness [6, 11, 14]. In non-infectious locomotor disorders in fattening pigs, gilts and sows, an improved lameness score was observed 24 h after treatment with meloxicam during gait and at rest [31]. After inducing lameness, meloxicam and flunixin led to improved leg loading and movement in sows [32]. Moreover, the severity of lameness decreased, but one week after the induction of lameness, it had disappeared [33, 34]. Sows affected by spontaneously occurring lameness and treated with meloxicam had longer standing times after feeding than those in the control group [14]. Pain therapies are reported to normalize the behaviour of lame sows [25]. A survey revealed that, if lame pigs were treated, Metacam was usually selected as the medication, but only a quarter of the farmers initiated treatment [26]. Meloxicam was named the most popular painkiller according to this survey.

### Skin ulcers and decubitus symptoms

‘Ulcer’ refers to an external skin trauma that mainly develops from ‘top to bottom’ and may even affect underlying bones [35–38]. In pigs, ulcers result from lying for a longer time in an unchanged posture, which compresses blood vessels supplying the skin and results in insufficient blood circulation, cell death, and necrosis [39–41]. This kind of ulcer is classified as a *pressure ulcer* or *decubitus* and mainly develops in areas of the body, where bones are hardly padded by muscles or subcutaneous fat and thus are compressed by a hard floor. In general, ulcerative skin lesions can develop in many regions, such as the limbs, tail, flank, udder, legs or ears [38, 42, 43]. Most attention, however, is directed to shoulder ulcers located above the *tuber spinae scapulae*, often initiated by longer lying during farrowing [35, 44, 45].

Irrespective of the location, most of the papers selected for this review did not focus on pain due to (shoulder) ulcers (for an example, see [35]). Topics address developing improved clinical detection scores [35, 46], increasing knowledge about behavioural responses and risk factors [44, 47], or assessing prevention [44] and treatment options [48, 49]. Moreover, a review of epidemiologic and forensic aspects [38] and knowledge about causes, prevention and treatment have been generated [39]. In view of these findings, it may not be surprising that a clear definition of pain and related processes [to be expected or assessed] is often missing. It can be inferred from previous studies that tissue damage and inflammatory processes typical of ulcers are related to acute pain [35, 38, 50, 51]. In this respect, one previous paper elaborated on acute phase proteins [haptoglobin and albumin, among others], which appear to develop in correspondence with bilateral shoulder ulcers [48]. On the other hand, chronic pain may occur given that neuromas can develop over time [35, 51]. Moreover, many papers argue that ulcers are painful for pigs because they are analogous to decubitus in humans, which can also be highly painful [35, 45, 48, 52, 53].

To assess the effect of (shoulder) ulcers on pigs, observers generated scores for lesions and/or for behavioural aspects. The scoring system used in these papers is not standardized but follows a similar pattern (for an overview see [35]). According to the tissue involved, for example, lesions with a score of 1 affect only the epidermis, while lesions with a score of 2 also affect the dermis. Lesions with a score of 3 affect all skin layers, including the subcutaneous tissue, and lesions with a score of 4 affect the entire skin and the underlying bone [35, 40]. Pain is likely to occur even with superficial layers, so findings such as size, scab, and wall alterations should be staged with regard to a histopathological score to avoid underestimation of clinical signs [35, 40].

Further clinical signs address the adaptation of behaviour [54]. Results indicate that even sows with moderately sized shoulder ulcers [3 cm in diameter] exhibit changes in behaviour, such as reduced lying time, increased frequency of postural changes, increased standing and reduced nursing frequency [54]. This behaviour appears to be both a response to pain and a way to protect against an exacerbation of the lesion [48]. Another change in behaviour is increased rubbing against fixtures of the farrowing crate [54], which may even be invoked by palpation of the shoulder to discriminate the reactions of affected and non-affected sows [51]. Moreover, a correlation between pain-associated reactions after palpation of shoulder ulcers and the depth of the lesion has been proven [55].

Although the results speak in favour of pain sensation in pigs due to shoulder ulcers, a study has recently outlined the question of whether (pain in) early stages of shoulder ulcers can be deduced from changes in routine behaviours [47]. Indeed, behavioural changes corresponding to early tissue changes, such as skin reddening, might be subtle. Detection of subtle changes requires a dedicated study design (in terms of time points in relation to farrowing (or previous ulcers), observation time, frequency, sample and effect size apart from a treatment-effect protocol). To the knowledge of the authors, no study elaborating on these points has been published thus far. In this regard, practitioners should carefully evaluate predisposed locations and consider providing pain relief in cases of initial skin changes even if changes in behaviour may be subtle or not obvious during examination.

To elaborate on the age or extent of chronic lesions, studies involving histopathological examination of skin ulcers can be considered [35, 38]. Concerning neural tissue, traumatic neuromas appear not only in shoulder ulcers affecting deep skin layers but also in ulcers affecting only superficial layers [35]. Moreover, traumatic neuromas have been found in shoulders with evidence of healed ulcers [35, 51]. It is commonly accepted that nerve damage may lead to increased activation of peripheral nociceptors and central neuronal excitability, including peripheral and central sensitization [56]. Moreover, neuroma formation is likely to initiate chronic neuropathic pain [57]. These changes contribute to post-injury hypersensitivity, which is measured as hyperalgesia and allodynia [51, 58]. Hence, a pig that has ulcers in predisposed locations should be examined more frequently for pain-related behaviour and should be considered a candidate for pain relief treatment even if no (acute) compromised skin is visible.

In summary, these findings reject the assumptions that nerves in damaged tissues basically cease [59] and support that both inflammatory and neuropathic pain can occur due to skin ulcers and cause pain in pigs. To emphasize this point again, painful changes in tissue may occur even before they are clinically visible to the observer, and these changes may persist after clinically visible signs have disappeared [35, 46, 51, 60]. Therefore, pigs should be carefully examined and treated for welfare concerns as early as possible, as outlined below.

Therapy for shoulder ulcers comprises the local application of zinc ointment [49], mãnuka honey, or local sprays [46] or the protection of the affected skin from soft rubber mats [44, 48, 49]. Pain alleviation via NSAIDs should be considered for shoulder ulcers affecting the dermis and certainly for those affecting deeper skin layers [61]. However, pain relief without accompanying treatment [effective decompression, early weaning] may have a contradictory effect [48]. If no pain alleviation is possible or no healing can be achieved, pigs with deep ulcers may need to be euthanized. Euthanasia should be considered when the underlying bones are affected. To avoid reaching this stage of ulceration and secure timely euthanasia in such cases, future studies should elaborate on the timeline of ulcer stages with regard to the appearance of chronic pain on the one hand or valid healing signs on the other hand.

Studies on healing published this far have shown that skin lesions are present in the majority of shoulder ulcers in sows for at least 2 to 3 weeks and that superficial healing usually occurs within a few weeks after weaning. The prominent type of healing is secondary healing [62]. However, the chance of developing chronic pain initiated by traumatic neuroma [51] and an enhanced risk of developing a shoulder ulcer [45] again show that healing is often limited by the disappearance of the visible skin lesion. Information about the healing of ulcers in locations other than the shoulder is not available to the authors.

When considering the influence of treatment on the healing of shoulder ulcers, attention needs to be devoted to the fact that ulcers rarely develop alone. In this respect, several studies have addressed risk factors such as lameness, a low body condition score or the development of shoulder ulcers during previous farrowing [39, 44, 63–65]. Climate conditions, such as moisture in crates and changes in floor types before farrowing, also appear to play a role [46]. Hence, when assessing the chance of healing or generating a treatment protocol, these factors need to be considered. If underlying causes are neglected (such as a lameness), secondary ulcers in unexpected regions may develop [such as over the ala of the ilium, the head of the femur, the elbow and various locations of the distal leg; see (Additional file S2 for assessment in stable (right side) and Additional file S3 for secondary ulcers (left side of the same pig)), or ulcers in typical locations may develop (Additional file S4).

In summary, shoulder ulcers evoke several pain mechanisms, and an appropriate treatment protocol is needed that may go beyond the consideration of pain relief or rubber mats alone. For detection, examiners need to assess skin and shoulder ulcers in pigs carefully and look for ulcers in unexpected regions when locomotion is impeded or when the body condition score is low.

### External hernia

Hernias are protrusions of tissues or organs (or both) through body walls within [internal hernia] or outside the body or compound (external hernia) [66]. Multiple hernia types are defined, and they can appear due to natural openings (defects), trauma or lesions of surrounding tissue, such as skin or muscles [66–68]. Umbilical hernias in pigs, however, have attracted the most attention because they have consequences for transport and slaughter [66]. Topics about umbilical hernias in reviewed papers address behavioural and pain indicators [66], genetic factors [69] and surgical aspects [75, 76].

Concerning pain sensation in general, umbilical hernias with intact skin are not necessarily painful [62]. They may cause abdominal pain or discomfort [69], but smaller umbilical hernias without intestinal incarceration or ulcerative skin lesions are likely not painful for the pig. As an indicator of pain, the results elaborate on reduced lying time [70] and the willingness of pigs to engage in locomotor and social activities [66]. The latter study revealed no differences between pigs with and without umbilical hernias. However, skin lesions in pigs with umbilical hernias were not reported, and observations were subject to variability. Therefore, findings on the willingness to engage in activities as an indicator of pain due to umbilical hernias needs to be interpreted with caution. From the perspective of the authors, a promising behavioural indicator for pain measurement in pigs, however, might be the time a pig is lying in sternal recumbency, as this position is associated with maximum pressure on the hernia, particularly in hernias where the intestine cannot be repositioned into the abdomen. Painful alteration of the hernia will likely reduce the duration of lying in sternal recumbency, which is usually favoured by pigs.

Additionally, pain is very likely to compromise animal welfare when ulcerative skin lesions on the outpouching are present [38]. Ulcerative skin lesions on hernias frequently start to develop from the ventral aspect and are considered painful [38]. In fact, they are the most frequent complication that lead to euthanasia of pigs affected by umbilical hernia [71]. The risk for skin ulceration is positively associated with the size of the hernia and is increased in hernias where the intestine cannot be replaced into the abdomen [71]. Thus, assessing hernias and accompanying conditions is important for determining further development (also in [66]). Another case in which pain is very likely to occur is the type of pathological condition causing the hernia [70]. The typical pathological conditions of hernias include strangulation of the hernia content with insufficient blood supply, obstruction or incarceration of the intestine and adhesions to the intestine or omentum [72, 73]. All these complications are associated with severe pain in humans [74], and it is reasonable to assume that this also applies to other species [72].

Surgical repair is an option for the treatment of umbilical hernia [75, 76], and related translational studies have been published [77]. However, as criticized in translational studies, pain relief is often not reported even though pain may be observed (also see [76, 77]). Like for ulcers in general, pain alleviation should be considered for hernia lesions affecting the dermis, those exceeding a medium size (e.g., a diameter of 2 cm) and, certainly, for deeper skin layers. When an ulcerative skin lesion on a hernia exceeds medium size or affects the subcutis (Additional file S5), healing within an acceptable time is unlikely, particularly when the pig is kept without litter, and euthanasia prevents further pain and suffering.

Concerning healing, the general processes of the pathology of hernias have been well researched [67, 68], and umbilical hernias tend to maintain their basic conditions over time [66]. For a second aspect, namely, the knowledge about the genesis and healing of invagination and adhesions or incarceration and secondary injuries of (umbilical) hernias, publications are rare. However, whether ulcerative skin lesions on hernias result mainly from damaging behaviour or whether the healing of skin lesions is compromised by the pressure applied to protruded organs has yet to be studied. In this review, several papers reported on post-surgery care [75, 76]. In addition, studies on other external hernias, the reporting of pain and the treatment of hernia-associated skin injuries in pigs are neglected in the literature. Moreover, studies addressing umbilical hernias, such as the transcriptome analysis of Souza et al. [69], do not discuss implications for pain. The study suggested that gene candidates for umbilical hernias may also indicate expression profiles where chronic pain occurs. However, it remains unclear what conditions the pigs and hernias were in (e.g., inflamed, ulcerated) and whether gene-related inferences about the presence of pain could be drawn.

### Biting lesions

Biting lesions caused by damaging behaviour of other animals in the group occur on the tail, ear, flank and vulva. Since there are various definitions for biting behaviour, ranging from incidental chewing to forceful attacks [78], biting was defined as any biting behaviour of pigs leading to skin damage for this review.

#### Tail biting

Tail biting is a frequently observed abnormal behaviour in domestic pigs and is closely associated with welfare and economic concerns [79–82]. Lesions can range from minor bite marks to loss of the entire tail [83]. Tail biting and the resulting lesions are connected to increased pain in pigs [81, 84] and are linked to local, secondary and systemic infections [79, 83]. In this regard, this topic is well researched, especially given the questions associated with damaging management procedures such as tail docking.

It has been suggested that tail biting is a redirected behaviour with a background in unfulfilled exploratory behaviour [85]. Nevertheless, tail biting is multifactorial, with several other factors having an impact on development [86, 87]. Despite the high research interest, neither standard terms for the act of biting [such as caudophagy, chewing, fanatic attacks, cannibalism] nor final theories explaining the motivation of pigs to engage in damaging behaviour are commonly applied or defined [78–80, 88–90].

Tissue damage ranges from superficial bite marks to bleeding or encrusted lesions, necrosis, abscesses or even loss of the tail body; this damage can include a loss of the entire tail as well as injuries to the tail root and the surrounding tissue. Repeated biting results in the coincidence of acute and chronic tail lesions, as some pigs are bitten multiple times [91]. According to the degree of damage, (histo-)pathology reveals invasion of inflammatory cells and development of granulation and scar tissue among common developmental stages [57, 88, 92]. The histopathology of tissue injured by biting has been described in several studies for pigs with docked and non-docked tails [57, 92].

Although undocked pigtails are likely to differ from docked tails in neuroanatomical terms, the latter can also be bitten. When discussing pain in this respect, one should consider the proliferation of neurofilaments, including neuromas, in tail tips as well as (pain/inflammatory) gene adaptation in dorsal root ganglia [92, 93]. While neuromas are not necessarily painful when induced by tail docking [92, 94], painful conditions after amputation are associated with coexistent scar tissues, abscesses, haematomas or osteomyelitis [95], conditions that are likely to occur in tails with severe biting lesions.

“Tail biting causes wounding of the tail, as well as amputation of part of, or the entire tail, which surely is painful for pigs” [89, p.144]. Pain caused by tail biting thus relates to acute pain, and the stimulus can be repeated when victims are bitten again [89, 91]. Inflammation and infections are often associated with the overall condition of pigs with bite injuries [79, 96], as are signs of chronic stress [89, p. 155].

The tail posture has been examined for its use as an early predictor of upcoming tail biting [97–99]. However, recent studies indicate that a hanging or tucked-in tail posture does not indicate early damage [86, 100]. A hanging tail posture is indicative of tail wounds but not of minor damage, such as bite marks [86, 100]. Inflamed wounds at feeding are significantly associated with hanging tails [86]. Nonetheless, hanging or even tucked tails can be found in pigs with no visible signs of damage [97] and may also be related to stress [86]. In general, specific postures in animals often cannot be assessed alone but need to be interpreted in combination with the entire body posture or behavioural examinations [86].

Other studies have evaluated the feeding behaviour of bitten pigs under pain relief such as ketoprofen [101]. It was found that before the onset of biting, the feeding frequency was reduced, and pain relief, as provided by the applied concentration, chosen signalling pathway or standalone treatment, seemed to have little effect on the return of feeding behaviour prior to onset [101]. More research is needed to clarify whether ketoprofen does not resolve stress or restlessness, a behaviour discussed earlier in this article as a sign of discomfort or pain (also see [96]). Another reason might be that other compromising conditions exist that impact feeding behaviour [102].

In addition to behavioural indices, biomarkers can be used to assess painful conditions in pigs [1], and several studies have evaluated this topic with respect to tail biting. In general, studies included in this review revealed that inflammatory marker levels tend to increase in bitten pigs [96, 103]. However, further research is necessary to determine whether higher or more systematic inflammatory biomarkers are associated with tail bites or severe damage [103, 104]. This knowledge is crucial for discussions about healing processes as well as the exacerbation of diseases.

In general, the healing of tail injuries [and pertaining to an outbreak of biting] can be traced macroscopically. In acute wounds, the blood appears fresh and bright red, while in older healing wounds, the blood becomes sticky, dry and dark [105, 106]. In the future, progressive dry tissue necrosis with eventual partial or total loss of the tail may occur within a few days [107]. While the initial leaking secretions keep the tail constantly moist for healing, they can also attract bacteria and trigger inflammatory processes [108]. The risk of infection of other organs via the bloodstream, lymphatic system and cerebrospinal fluid is almost always present in tail wounds. In addition to the skin, the tail muscles and tail vertebrae are often affected [88]. In addition to local infection, systemic spread of bacteria may lead to arthritis or abscess formation in the spine or lung [96, 109]. Despite these risks, reliable conclusions about mortality rates in tail-bitten pigs are not easy even though it can be assumed that tail biting leads to significant losses [80, 89]. Thus, tail-bitten pigs need early and thorough examination to determine which therapy and treatment are most appropriate.

In cases of tail injuries, local treatment, such as the application of chlortetracycline (CTC) spray [110] in combination with an analgesic, may be beneficial. Ketoprofen (mind the discussion above) or meloxicam (according to the experience of authors) may be assessed for a fit of treatment in a particular case. If signs of inflammation are present (swelling, redness of the tail], additional systemic antibiotic treatment is indicated [107, 112] to treat the local infection and prevent secondary infections likely induced by bacteraemia [96, 111]. Moreover, pigs with severe tail injuries involving partial or total loss of the tail should be moved to a hospital pen, and identified aggressor animals should be removed from the group [112]. The chances of recovery from tail biting are good if the animals are treated early. In a previous study, a healing rate of 89% was achieved through the provision of additional enrichment materials and removal of the perpetrators [112]. However, if an infection has occurred, the prognosis for the animal is poor [113].

#### Flank biting

Flank biting can occur due to damaging behaviour similar to tail and ear biting [99, 114, 115]. The pathology of biting lesions are similar in histopathological terms. Hence, depending on the intensity, depth and involvement of bacteria, findings range from superficial epidermal lesions to deep ulceration [116].

In general, studies dedicated to flank biting alone are rare [99]. Following one study in the review, the effects of flank biting on pigs can be described by scoring the lesion (size, freshness, severity) and assessing behavioural indicators. Compared to tail biting, this study found that the tail posture is not a reliable indicator of pain or distress due to flank biting. However, the occurrence of flank injury tended to be associated with the severity and severity of certain tail injuries (p.11).

Apart from the reviewed publications, deep lesions and ulcerations of flank lesions should be considered painful, as discussed in the previous chapter. However, findings about pain-specific behavioural indicators for flank biting, treatment or healing processes have yet to be published and discussed in future studies.

#### Ear lesions

Ear biting refers to the oral manipulation of ear tips or bodies and can result from damaging behaviour [115]. An associated pathology is porcine ear necrosis (PEN), which can result from initial bite lesions, although other aetiologies are discussed [117]. This chapter subsumes PEN and ear biting as ‘ear lesions’, as both entities represent spontaneously occurring injuries, and the terminology used in related papers is often vague [117].

Furthermore, the [histo-]pathological characteristics of ear lesions are a well-known problem in pig production and have long been assessed [116, 118]. In addition to developing like other bite lesions [see previous chapters], blood vessel occlusion induced by bacterial toxins is considered to be the main cause of epidermal damage and necrosis [117]. Irrespective of the cause, mild and superficial lesions to severe necrosis and substantial loss of ear bodies may occur.

Although tissue damage and necrosis in body parts are likely to be accompanied by pain, none of the papers discusses this subject in particular. Among the papers examining ear biting, however, one study developed an ethogram for biter and bitten pigs and studied behaviours such as pain-related vocalization [115]. The findings from this study suggest that ear pulling, head shaking and, to a lesser extent, quick bites invoke greater pain, as indicated by the avoidance behaviour and screaming of the bitten pig [115, p. 34]. Moreover, a study on behavioural and physiological responses to damaging procedures was integrated [119]. The findings from this study suggest that a combination of head shaking, ear scratching, shivering and grunting indicates pain after ear tagging or notching (p.92).

Apart from behavioural parameters, studies concerning ear lesions often use scoring systems similar to those used for tail lesions. For example, clinical or histological examinations concerning the size, freshness, depth or loss of the ear surface have been performed (for an overview see [117]). Interestingly, none of the scores or stages of severe lesions were discussed in relation to pain.

In comparison to the treatment of such lesions, the healing processes and prognoses depend on the particular lesion state and are rarely discussed. In general, stages ranging from re-epithelization with intermediate clinical signs of crusting and leakage similar to tail bites to a final, total loss of the ear are possible [117, 118].

Treatment of ear-bitten pigs depends, of course, on the stage of the lesion. Separating pigs to a hospital pen, providing materials to reduce damaging behaviour and mitigating risk factors are generally advised. Interestingly, providing antibiotics is a discussed strategy due to the assumed involvement of bacteria in the onset or exacerbation of a lesion [117]. Although this topic has not been discussed previously, pain relief for pigs with substantial ear lesions should also be considered. However, further research is needed to examine the influence of medications such as NSAIDs on the healing process and welfare of pigs with ear lesions [115, 117].

#### Vulva biting

Vulva biting is a behaviour observed in group-housed sows and is often associated with factors such as frustration, competition for food and limited access to resources [120]. It serves as a commonly used indicator of sow welfare.

The swelling of the vulva in late pregnancy increases vulnerability to attacks [121], leading to enhanced occurrence during this period [122]. Once the vulva is bitten, the increasing swelling, discolouration, and potential presence of blood and pus attract further attention, thereby exacerbating pain [120]. Sows show signs of self-protective behaviour by sitting down in feeding queues, likely to avoid being bitten by the following sow [123] or by keeping the time to feed at the trough particularly short [124], while bitten sows react very sensitively and try to flee the situation [120]. Taken together, these findings indicate that vulvar bites cause considerable pain to sows. However, in terms of pain alleviation, the significance of vulva biting lesions has often been disregarded, and it is not unlikely that this is caused by a misunderstanding. An increase in the nociceptive threshold has been shown during late pregnancy and parturition not only in women but also in sows [125], perhaps as an endogenous defence against the pain of parturition [126]. This mechanism might facilitate pain alleviation during parturition, but there is no evidence that vulvar lesions induced by biting are generally not painful.

The severity of the wounds can vary, ranging from fresh wounds to old scars, and complications such as secondary infections can occur [127]. Skin-perforating lesions showing signs of inflammation are likely painful for affected sows. In severe cases, vulva biting can result in extensive scarring and disfigurement, possibly causing considerable pain during subsequent farrowing [128]. Timely identification and removal of affected sows from the group, along with appropriate analgesic treatment, are necessary to minimize pain and further damage [122].

### CNS (meningitis)

Meningitis is a common disease in pigs and is considered to cause pain [6]. Among the reviewed papers, only one addressed pain in CNS-related diseases [129].

In general, *Streptococcus suis* is the most important pathogen that generates meningitis worldwide, and *S. suis* infections affect 5- to 10-week-old pigs in most cases. Clinical signs of this infection may include septicaemia and acute death, meningitis, polyarthritis, polyserositis, and valvular endocarditis [130, 131]. Although most weaned piglets carry *S. suis* strains, few carry virulent strains capable of inducing the disease. In peracute cases, pigs may die without any preceding clinical signs. Otherwise, pigs may exhibit incoordination and adoption of unusual postures in early stages, which soon progress to inability to stand, paddling, opisthotonus, convulsions, and nystagmus. Other clinical signs may also be observed [132], but early recognition and immediate parenteral treatment with an appropriate antibiotic with or without an anti-inflammatory agent maximizes pig survival [133].

As *S. suis* is also well known as a zoonotic agent, assumptions about pain related to its meningitis can be supported by manifestations in humans. Clinically, headache, fever, vomiting, nervous disorders, and later hearing loss can be observed most frequently [134]. According to different systematic reviews, fever, headache and neck stiffness also appear to be the most prominent clinical signs [135, 136]. Headache certainly presents a sort of pain, but as shown in a case study of a Persian cat, severe mid-lumbar back pain and extreme reluctance to move were the only abnormalities on physical and neurological examinations [137]. Hence, pain induced by *S. suis* infections may be obvious not only in pigs with lameness but also in those with meningitis [6], as described in human medicine and other species.

In pig practice, anti-inflammatory drugs are recommended for the treatment of meningitis because in addition to their anti-inflammatory effects, they can reduce pain and have antipyretic effects [138]. However, the effects of treatment with analgesics in cases of *S. suis* infection in pigs have rarely been documented. A study evaluated the effect of buprenorphine treatment in an *S. suis* infection model in pigs [129]^1^. The intravenous administration of *S. suis* to 6-week-old piglets led to severe disease in approximately 50% of the animals. Suppurative meningoencephalitis and arthritis as well as fibrino-suppurative endocarditis were the main findings at necropsy. For pain scoring, the following parameters were assessed: feed intake, lameness, movement time, get-and-scare up, pain vocalization, and behaviour (fresh, damped, listlessness, central nervous disorder [tetanic spasm, opisthotonus, convulsion]). Additionally, special signs (kyphosis, tremor) were evaluated. The administration of 0.05 mg of buprenorphine/kg for 5 days i.m. every 8 h *post infection* did not prevent high clinical or pain scores in affected animals and did not result in substantially lower mean clinical or pain scores. Hence, the chosen protocol of buprenorphine application does not prevent severe distress or pain in this infection model [129].

### Respiratory tract

Infections of the respiratory tract are among the most common health problems in pig husbandry. In addition to the general negative impact on pig health and welfare, respiratory tract infections can cause reduced feed intake, decreased daily weight gain and increased mortality. Symptoms of respiratory tract infections in pigs include reduced general condition, fever, dyspnoea, painful breathing (sometimes in the dog sitting position), coughing, sneezing, nasal discharge, cyanosis, reduced feed intake [anorexia], reduced daily weight gain, and, in the worst case, death [139].

Respiratory infections not only impact the lungs but also affect the trachea and nasal cavities. Pneumonia and/or bronchopneumonia and pleurisy are the main findings in this context in the affected pigs at slaughterhouses [140].

Even though respiratory tract infections are common in pigs worldwide, there is a lack of literature concerning pain caused by respiratory tract infections in pigs at every age. As stated by Pessoa et al., respiratory tract infections are likely to have a significant negative impact on pig welfare ([140], p.1), although such an effect is rarely considered in the literature. Indeed, studies on indicators of actual pain followed by respiratory tract infections and the potential impact on pig welfare are missing.

This is all the more surprising because, in a survey by Ison and Rutherford, farmers and veterinarians rated respiratory diseases in pigs as painful and scored the expected pain with 5.1 on an 11-point scale [26]. The recognition and management of pain was assessed as important in this study, and many participants in the survey expressed an interest in identifying pain in pigs as well as the treatment options available.

However, information on pain in relation to porcine respiratory diseases is limited to studies on porcine pleuropneumonia caused by *Actinobacillus (A.) pleuropneumonia*. For instance, infections induced by *A. pleuronpneumoniae*, which can cause necrotizing pneumonia, fibrinous pleurisy, pulmonary oedema, and dyspnoea, are associated with severe thoracic pain [141]. Therefore, careful monitoring and early intervention involving euthanasia are recommended to avoid unnecessary suffering [141]. Furthermore, Swinkels et al. showed the beneficial effects of Ketoprofen in addition to antibiotics on recovery rates and feed intake in pigs infected with *A. pleuronpneumoniae* [142].

Despite the lack of published data on pain associated with pulmonary diseases in pigs, pain is undoubtedly one of the cardinal symptoms of any inflammatory response and thus also of respiratory tract infections. Therefore, it can be assumed that pigs experiencing respiratory tract infections suffer pain.

In addition, noxious gases released from pig faeces, such as atmospheric ammonia, can irritate even healthy respiratory tracts. Poor air quality increases the risk of respiratory diseases by irritating the epithelium and even causing cilia loss/function [143]. If given the choice, young pigs would more likely choose to stay in areas containing no atmospheric ammonia than in areas with even lower concentrations (10–20 ppm) [144]. Furthermore, Jones et al. found that pigs left areas where ammonia was present after approximately 35 min [144]. Because of the lack of immediate aversion, the authors concluded that not the odour of ammonia caused the pigs to leave these areas but rather the discomfort caused by the gas.

While a respiratory tract irritated by (high) levels of atmospheric ammonia is by definition not a disease, it should not be underestimated concerning the resulting discomfort and even damage and pain and the potential infections of the respiratory tract that could follow.

### Gastrointestinal tract

Pain resulting from various gastrointestinal diseases in humans is an important research field in which the species pig is widely used as an animal model. Some information about pain in gastrointestinal diseases in pigs can therefore be deduced from neuroanatomical and physiological findings in these translational studies. The intestinal nervous system in humans is mainly autonomic but is in part controlled centrally by extrinsic innervation via three pathways: parasympathetic and sympathetic efferent innervation and sympathetic afferent innervation. It is known that pain stimuli from the stomach and intestine are conducted by afferent nerves to the brain [145].

In contrast to studies in humans, pain assessment studies of gastrointestinal disorders in pigs are rare. In most studies, important animal-based indicators, such as behaviour and vocalization, are used to assess distress without differentiating pain as a stressful condition [146]. In gastrointestinal diseases, behavioural scoring for pain assessment can be considered more meaningful than vocalization, which has been used for acute pain combined with extreme stress, e.g., during surgical castration [147, 148].

Rectal prolapse is a specific disorder of the gastrointestinal tract that can be differentiated by its length and obvious injury to the mucosa. Due to acute painful events, when the non-physiologically exposed mucosa is injured or destroyed, pain assessment might be easier than in other gastrointestinal diseases. Several stress biomarkers, including cortisol and salivary alpha-amylase, have been used to assess pain during rectal prolapse to validate a modified pain perception protocol published by Morton and Griffith [6, 147]. A score from 0 to 20 was based on the patient’s appearance and body condition score, clinical signs and behaviour without any stimulus and response to external stimuli [149]. A combined score equal to or greater than 5 indicated not only pain but also distress and discomfort. Pain, distress and discomfort were recorded for pigs with a prolapse length of 6+-2.5 cm but not for pigs with smaller prolapses (3.0+-4.3 cm). The scoring outcome was related to several biomarkers [147].

In other gastrointestinal diseases, pain assessment can be biased by inappetence and other disease-related behaviours [6]. Gastrointestinal diseases were scored as moderate by veterinarians and farmers, ranging from 4.5 to 5.6 on a 10-point pain scale based on their experience [26]. Assessment of pain relies mainly on behavioural characteristics [150]. Behavioural indices for the assessment of acute pain have been described for piglets after intra-abdominal injection, which can cause abdominal pain. Typical pain-related behaviours such as rubbing the abdomen against the floor and huddling (i.e., tucking three or more legs under the body) have also been observed [119, p.89]. In addition, control pigs and treated pigs differed with respect to being awake without moving, as abdominal pain seems to be quite subtle, “awake inactive behaviour”, (119, p. 91). According to the experience of the author team, metamizol may be assessed in a given case given its spasmolytic characteristics [198, 199].

Gastric ulcers in sows and in finisher pigs are important welfare issues in swine production and occur at varying rates among countries and studies [151]. In finisher pigs with gastric ulcers, behavioural changes were observed and interpreted as pain-related behaviour. Pigs with gastric ulcers spend significantly more time walking and standing than their pen mates and tend to rest less and avoid lying on the left side of the body [152]. In contrast, behavioural observation of fattening pigs two days before slaughter and inspection of gastric lesions in the respective pigs resulted in more lying in contact with other pigs, more manipulation of pen mates and longer eating times in affected pigs [153]. In translational medicine, the “acetic acid ulcer model” is well established for studying interventions for gastric ulcers in swine [154]. The results obtained in this model revealed the impact of antral ulcerations on the intramural nerves responsible for the function of the pyloric sphincter. In humans, this painful disease is characterized by a malfunction of gastric emptying [154]. In this experimental pig model for gastric ulcers, neuronal responses were studied. The expression of the neuropeptide galanin, which is widely distributed in the gastrointestinal tract and modulates the enteric nerve response, among other biological functions, was also examined in inferior vagal neurons in pigs [155]. Neuropeptides such as galanin are synthetized by primary afferent neurons and are involved in visceral pain signalling. In swine with experimentally induced gastric ulcers, galanin expression was significantly greater than that in healthy control animals, supporting the assumption that gastric ulcers are painful [155–158]. Abdominal pain is a symptom of various anatomical and functional gut alterations, such as intestinal inflammation, partial blockage and gut distension. Pain is considered the most important symptom in 50–70% of patients with inflammatory bowel disease [159]. It is also known that persistent changes in afferent neurons due to sensitized sensory pathways and despite resolved inflammation can lead to persistent pain [159]. Enteric inflammation in swine due to various pathogens can lead to pathomorphological inflammatory and functional changes similar to those in humans; thus, the occurrence of abdominal pain can be hypothesized, at least in severe cases [160]. The ileocecal valve is innervated by dorsal root ganglia neurons, and specific bacterial lipopolysaccharides can influence the neurochemical reactivity to neuropeptides at this location [161]. It can therefore be hypothesized that swine infectious enteric diseases impact neurophysiological mechanisms and therefore pain signalling.

### Urinary tract infections

Inflammatory processes in the porcine urinary tract of sows are mostly caused by facultative pathogens such as *Escherichia coli*, streptococci, staphylococci, *Proteus* species, *Klebsiella* species and *Trueperella* (*Actinomyces) pyogenes* [162, 163, 196]. Lesions due to nonspecific urinary tract infections [UTIs] are usually restricted to the urinary bladder and cause only mild clinical signs. Contamination and ascent of the urethra by faecal microbiota is possible but more likely in females than in males.

The clinical signs in sows with cystitis include urinary changes in most cases. Some pigs urinate in small quantities with straining or be observed in a dog-sitting position [162, 164]. General signs of illness become apparent if cystitis is followed by ascending infection to the kidneys and the development of pyelonephritis, resulting in uraemia [165–167].

The clinical symptoms may depend on the involved pathogen. For example, generalized infection of the urinary tract is often caused by *Actinobaculum suis*, which is carried by boars and can be transmitted to sows during mating. Clinical signs may develop 2–3 weeks after mating or may be delayed until farrowing. Affected sows or gilts may die suddenly or be found ill, depressed, or thirsty with hunched backs. Haematuria is the main sign of the acute phase, together with frequent and painful voiding and dysuria. Later, the affected animals are uraemic; they pass bloodstained, purulent urine with or without vulval discharge; and they exhibit inappetence and weight loss. Typical cases exhibit the following symptoms: hypothermia below 38 °C, a heart rate greater than 120, painful abdomen, polypnea, cyanosis, ataxia, and more rarely generalized tremor. Moreover, clinically affected sows frequently die from renal failure [165, 167–169].

Regarding behavioural changes, pain due to UTI can be expected, especially if haemorrhagic cystitis and pyelonephritis are present. In the review, however, no studies elaborating on pain in relation to UTI in pigs were found. This is astonishing since much about the pathophysiology of the urinary tract, including the neuronal patterns of pigs, is known and appears to perform similarly to what has been done in humans [170, 171].

The symptoms of acute cystitis in humans are similar and include dysuria with or without increased frequency, urgency, suprapubic pain, cloudy urine, or haematuria [172–175]. Dysuria is defined as the sensation of pain and/or burning, stinging, or itching of the urethra or urethral meatus associated with urination [176]. Hence, it must be considered that pigs with bacterial cystitis can also experience some pain at least when urinating.

In addition to the lack of studies about pain indicators related to UTIs, very little is known about pain treatment for cystitis in sows. In addition to antibiotic treatment, Stirnimann et al. recommended the administration of a spasmolyticum/analgeticum [Metamizole], as the results obtained for acute UTIs in sows were better than those obtained with antibiotic treatment alone [165, 166].

### Mastitis and teat lesions

Mastitis is an inflammatory process of the mammary gland and is caused by bacterial infections and/or traumatic teat lesions. Acute mastitis affects sows mainly within 3 days after farrowing and is accompanied by systemic signs (fever, reduced feed intake) and local signs of inflammation (oedema, skin congestion) [177]. Risk factors for the occurrence of teat injuries include the presence or absence of tooth resection in piglets, different methods of tooth resection, housing systems (especially flooring), litter size, piglet-related management strategies, milk supply and barren environments [178]. Interestingly, posterior mammary complex pairs are often affected, potentially due to sows injuring themselves with hind claws [179–181] or likely to have lower milk production in the caudal glands [182]. Piglets working harder at the rear udder due to lower milk supply might lead to more accidental biting. Additionally, the distinct nervous supply of pair number 7 could decrease their sensitivity to pain, resulting in slower pain reactions to injury [179].

The pathogen involved in mastitis may influence the set of clinical signs in a sow. In general, however, mastitis is considered to be a postparturient disorder complex, such as postpartum dysgalactia syndrome (PPDS) (formerly referred to as mastitis-metritis-agalactiae (MMA)), rather than a distinct disease entity (cf. [183]). This approach makes it difficult to define pain-specific behaviour, although mastitis in sows is generally considered painful by veterinarians and farmers [6, 26], similar to what is the case for cows [184, 185]. Regarding mammary skin lesions, histological findings and bacterial identification revealed dermal and subcutaneous pyogranulomatous lesions due to infection with *Staphylococcus aureus* and dermal abscesses associated with *Trueperella pyogenes*. The glandular tissue of the mammary was unaffected, which ruled out mastitis [179]. Another study found an association between mammary lesions and *Pseudomonas spp.*, primarily affecting the dermis with potential gland involvement. Inflammation is driven by lymphocytes via the IL-1β/IL-6 pathway, partly involving T cells [186]. Recent evidence also links IL-1β to inflammation and pain [187].

Sows in pain may exhibit various avoidance behaviours, including attempting to move away from piglets, restlessness, and alterations in posture, such as dog sitting or increased ventral lying patterns [188–191, 197]. They may also modify their maternal behaviours, potentially reducing nursing frequency and increasing aggression towards piglets [192, 193].

The prevalence of teat lesions may range from 3.3 to 19% and may not be detected by inspection alone [186]. Summarizing, it is striking that pain due to mastitis and teat lesions in sows are (still) only marginally discussed in the reviewed literature [183, 186].

For pain mitigation in mastitis, treatment with an NSAID (flunixin, meloxicam) combined with antibiotics and oxytocin is suggested to be effective at improving clinical conditions [194]. Although farmers may tend to treat severely diseased pigs [26], another study found that farmers rated mastitis to be the second most frequent reason for the administration of NSAIDs [30].

NSAIDs are rather effective at mitigating inflammation than pain; however, the authors generally indicate an overall improvement in the recovery of the animals during treatment [26, 183]. However, no further knowledge about the healing processes of mastitis and udder pathologies is available, although further study is needed to avoid related pain (cf. [186).

## Discussion and conclusion

The results of the review emphasize that pigs experience pain due to spontaneously occurring diseases and injuries, but systematic knowledge about this topic is scarce. On the one hand, there is a lack of publications about certain topics, such as flank biting. On the other hand, certain publications elaborate on topics that involve pain indicators, but discussions neglect the implications of results for pain identification, measurement and treatment. The last point is true for research in veterinary as well as human medicine generated for translational interest. Hence, one core implication of the review is that more systematic research on pain in pigs is needed for rare diseases (such as UTIs) and topics that were excluded in our reviews such farrowing in sows (focus on reproduction management). Another implication is that conducted research should involve standardized protocols to document, analyse and share results on pain detection beyond the project timeframe. Creating a standardized protocol is indeed another core implication of this review. The findings of this review suggest that a standardized protocol would comprise the observation of validated pain identification measures of different kinds (behavioural, biomarkers) over time and in relation to administered pain treatment. Based on a set of comparable studies, it will be possible to validate these assumptions and enhance the evidence about pain in pigs in the future.

With regard to evidence, it must be considered that the findings in this article originate from a scoping review without addressing the evidence level or risk of bias [3, 195]. Furthermore, the search strings contained exclusion criteria (such as the term “NOT”) which may have excluded relevant results.

In the future, systematic reviews weighting results based on evidence and quality of the sample size and statistics are needed once enough papers are available. Irrespective of statistical significance, the review and synthesis of publications show that pain in pigs due to spontaneously occurring diseases and injuries is often perceived, measured and treated to the benefit of individual pigs. It is imperative for veterinarians and farmers to assure that pigs do not suffer from unnecessary pain that can be relieved. The results of this study invite readers to reconsider in each patient whether pain and related indicators are present and how to resolve the condition to ensure the welfare of individual pigs.

## Electronic supplementary material

Below is the link to the electronic supplementary material.

Supplementary Material 1

Supplementary Material 2

## Data Availability

No datasets were generated or analysed during the current study.

## Footnotes

1 In Germany, the opioid analgesic buprenorphine is not licenced for use in pigs.
