# Supplementary material for: Part II: understanding pain in pigs—pain assessment in pigs with spontaneously occurring diseases or injuries
Source: Porcine Health Manag. 2025 Mar 12;11:13. doi: 10.1186/s40813-025-00420-1 (PMC11900645; doi:10.1186/s40813-025-00420-1)
Supplement: Supplementary file 1 — Supplementary Material 1 [file 40813_2025_420_MOESM1_ESM.docx]

# Supplements

## Details concerning the review method

For the review, a shared set of eligibility criteria was defined. Papers were included if accessible and peer reviewed or books (chapters) published between 2015 and end of March 2023 besides standard literature on pig diseases and work found through snowballing technique. Included languages are English or German. Concerning the topic, papers are included if they elaborate on the search terms in more than one sentence (buzz-words), i.e. contribute to the topic with descriptive or detailed in sight. The evidence level of selected papers is important, will be recognized and discussed in this study if needed. However, given the lack of published papers about the topic, the evidence level is not defined as a selection criterion.

The primary search database is VetSearch (EBSCOhost Research Database), which is a university organized search mask of the Stiftung Tierärztliche Hochschule Hannover. It searches through PubMed, AGRIS, CABI, Web of Science, SCIELO, SpringerLink, Wiley, ScienceDirect among other literature databases. To control the search process, the search was conducted in the database CABI searchRxiv (CABI Digital Library) as required by the journal to increase transparency of the process.

Since the search phrase PICO in human medicine does not apply to animals who cannot report their intervention, the search phrases are formed as a combination of: terms of animals ("pig*" OR "hog*" OR "porcine" OR "swine" OR "boar*" OR "sow*" OR "piglet*" OR "weaner*") ("pig*" OR "hog*" OR "porcine" OR "swine" OR "boar*" OR "sow*" OR "piglet*" OR "weaner*") AND the focus (“pain”) AND terms for specific diseases or injuries (topics). In German language, terms for animals were: ("*Schwein*" OR "Sau" OR "Sauen" OR "Eber" OR "Ferkel" OR "Absetzferkel") and for pain were: (“Schmerz* OR “Weh*”). However, several modifications had to be made so the whole phrases and terms for each chapter is outlined in the following sections.

## Review for locomotor diseases

The terms for the search of this topic in English were: (“lame*" OR "stuck" OR "la* down" OR " lack of coordination" OR "locomotor" OR "paralysis" OR "paresis" OR "hind limb*" OR "hindquarter weak*" OR "musculoskeletal" OR "movement" and added later on for search 2: "unsteady gait" OR "trembling" OR "arthritis" OR "fracture" OR "spine" OR "Spine abcess". To improve the output of findings, animal terms were searched in keywords and the rest in Abstract. In a second step, animal was searched in title and pain in keywords and rest in text which yielded 181 papers. Of both outputs, 46 papers were selected for screening and 19 selected for the review. Subsequent snowballing was needed to gain more results (n=12). In CABI searchRxiv, n=37 papers were found, but no one were selected. In German language, topic terms were: ("Bewegungsapparat" OR "Lahmheit" OR "Hinterhandschwäche" OR "Hundesitz" OR "Beinschwäche" OR "Festliegen" OR "Gelenkentzündung" OR "Arthritis" OR "Arthrose" OR "Polyarthritis" OR "Klauenverletzung" OR "Panaritium" OR "Stallklaue" OR "Afterklauenabriss"). All terms were search in text (n=27) and no paper was selected. In CABI searchRxiv, one result were found with given terms, even when looking for terms without specification, but the paper was not selected.

## Review for skin ulcer and decubitus

The terms for the search of this topic in English were: ("shoulder*" OR “ulc*" OR "decubital" OR "sore*") and in another search (“decubit*”). Initial findings of n=>100.000 (terms in text) without a proper fit required a specification of terms. Due to this, terms for the topic and animals were searched in the title and pain was searched in text, resulting in n=67 findings (without duplicates). Throughout the analysis, n=20 were retrieved for a closer screening and eight were selected for the review. Other combinations of terms in title, text and keywords did not yield better results. In CABI searchRxiv, the terms were searched without specification to increase results. Based on this, n=341 findings were found but no paper was selected for the review. In German language, topic terms were: ("Schulter*" OR "*Ulkus" OR "Ulzer*" OR "Dekubitus" OR "dekubital*" OR "Wund" OR "Wunde" OR "Wunden"). All terms were search in text (n=363 without duplicates). N=4 papers were gathered for a closer screening but none was selected for this chapter. In CABI searchRxiv, no results were found with given terms.

## Review for external hernia

The terms for the search of this topic in English were: ("outpouch*" OR "umbilic*" OR "hernia*" OR "stalk" OR "navel"). Terms for the topic and animals were searched in the title (pain in text) in a first step (n=32) and animal and pain in title (topic in text) was searched in a second steps (n=10). Other combinations in title, text and keywords did not yield better results. With the specification as outlined, n=7 papers were retrieved for a closer screening and three papers were selected for the review. In CABI searchRxiv, no papers were found even though terms were searched without field-specification. In German language, topic terms were: ("Hernie" OR "Nabel" OR "Nabelbr*ch*" OR "Umbilikal*" OR "Bruch"). All terms were search in text (n=85) but no paper was selected. In CABI searchRxiv, no results were found with given terms.

## Review for biting lesions

The terms for the search of this topic in English were: ("lesions" OR "lesion" OR "biting" OR "bite*"). Moreover, a field specifying the region ("tail*" OR "ear" OR "ears" OR "skin") and (“vulv* OR “flank” OR “aural”) was set as well as an exclusion NOT (“docking”) to manage initial results of n=>65000. Several combinations in title, text and keywords were assessed, including modifications that neglected regional terms such as in title (“bit*” OR “*lesion*”) with in title ("sensiti*" OR "nerve*" OR "neur*" OR "pain"). N=108 findings for topic and region in title (rest in text) and in a second step, topic in text and animal in keywords n=46, yielded best results. With the specification as outlined, n=20 papers were retrieved for a closer screening but none was selected for the review. Subsequent snowballing was needed to gain results (n=10). In CABI searchRxiv, n=641 results were found without field-specification but none was selected for the review. In German language, topic terms were: ("Läsion*" OR "*beißen" OR "*biss*") and the regions ("*Schwanz* OR "*Ohr*" OR "Haut") as well as (“Flanke”) and exclusion of NOT (“*Kupieren”) was set. N=12 findings retrieved but needed more searching for the exact terms “Schwanzbeißen” or “Ohrbeißen”. N=2 papers were selected for screening. In CABI searchRxiv, no results were found with given terms, even when looking for terms without specification.

## Review for CNS (Meningitis)

The terms for the search of this topic in English were: ("CNS" OR "central nervous system" OR "brain*" OR "mening*" OR "*cephalon" OR "spinal cord" OR "medulla" OR "spinal marrow") and terms excluded (NOT) were ("guinea" OR "woman" OR "women" OR "man" OR "men" OR "mice" OR "nursing" OR "patient*"). In a first step, animal and topic in title (pain and exclusions in text) yielded n=17 results. In a second step, pain was searched in title, animal terms in keywords (rest in text) yielding n=10 results. N=12 were selected for screening. Eventually, two papers were selected for the review. In CABI searchRxiv, no papers were found even though terms were searched without field-specification. In German language, topic terms were: („ZNS“ OR „zentrales Nervensystem“ OR „Hirn“ OR „Mening*“ OR „*zephalon“ OR “Rückenmark*” OR “Medulla*” OR “Mark“). All terms were search in text (n=51) and one paper was selected for screening. In CABI searchRxiv, no results were found with given terms, even without specification.

## Review for respiratory tract

The terms for the search of this topic in English were for search 1 ("breath*" OR "respiratory disease" OR „resp* organ* tract“ OR "respiration*") and added later on for search 2: ("pneumonia" or "lung inflammation" or "pneumonitis" or "pulmonary inflammation" or "serositis" or "pleur*"). To improve the output of findings, animal terms were searched in keywords and topic in title, the rest in text, resulting in 1 paper and 0 papers. In a second step, animal was searched in keywords and pain in keywords and rest also in keywords which yielded 39 papers. Of both outputs, 3 papers were selected for screening and 1 was selected for the review. In German language, topic terms for the first search were: (“Dypsnoe“ OR “Husten” OR “Flankenschlagen” OR “abdominal* Atmung” OR “Maulatmung” OR “Nasenausfluss”), for the second search (“Atem*” OR “Atmung” OR “Respiration*” OR “Dyspnoe”), for the third search (“Brustfellentzündung” OR “Pleuritis” OR “Lungen*” OR “Pneumonie”) with only one paper found in total. For the further search, the terms for pigs were neglected and second and third search were iterated, yielding 18 results. In CABI searchRxiv, the search was iterated but no additional results were found.

## Review for gastro-intestinal tract

The terms for the search of this topic in English were for search 1 ("mouth" OR "tooth" OR "teeth" OR "tonge" OR "maw" OR "gult" OR "pharyng*" OR "*sophag*" OR "gut" OR "stomach" OR "venter" OR "abdominal" OR "ileum" OR "ile*" OR "jejunum" OR "jejun*" OR "duodenum" OR "duoden*" OR "rectum" OR "throath" OR "intestin*" OR "gastrointestinal tract" OR "GIT" OR "rectal" OR "enteritis" OR "pulp*" OR "parodont*" OR "gingiv*" OR "proctitis" OR "diarrh*" OR "bowel" OR "gastric" OR "colon" OR "enteropath*") and added later on for search 2: (“gastric”, “bowel”). To improve the output of findings, animal terms were searched in keywords and topic in title, the rest in text. In addition the terms were excluded in text (NOT "pigment*" OR "board" OR "women" OR "woman" OR "men" OR "man" OR "mouse" OR "mice" OR "rat" OR "rats" OR "patients" OR "guinea") resulting in 1:74 papers and 2:8. In a second step, animal was searched in title and pain in subject and rest in text which yielded 28 papers. Of both outputs, 26 papers were selected for screening. In CABI searchRxiv, n=492 papers were found, 17 were screened but none was selected for the review. In German language, topic terms were: TX ("Mund" OR "Mundhöhle" OR "Zähne" OR "Zahn*" OR "Zunge*" OR "Rachen" OR "Schlund" OR "Hals" OR "Speiseröhre" OR "Magen*" OR "*darm" OR "GIT" OR "Gastrointestinaltrakt" OR "Ileum" OR "ile*" OR "Jejunum" OR "jejun*" OR "Duodenum" OR "duoden*" OR "Zwölffingerdarm" OR "Pharyn*" OR "*sophag*" OR "abdominal*" OR "Rektum" or "rektal*" OR "Bauch*" OR "Enteritis" OR "pulp*" OR "parondont*" OR "gingiv*" OR "proktitis" OR "Kolon" OR "Enteropathie" OR "Intestinal*") and later on “Kolon, Enteropathie OR Intestinal”. All terms were search in text (n=385) and one paper was selected for screening. In CABI searchRxiv, no results were found with given terms, even without specification. Another search 3 was conducted for the terms “("prolaps*" OR "stricture*") firstly by putting topic and pain in title first and secondly animal-terms in subject, pain in text and topic in title. This search yielded 46 paper and 4 were selected for review. In CABI searchRxiv, no results were found. In German terms, the search was reduced to a textsearch for “Proplaps” (n=9) and “Striktur” (n=8) but no paper was selected. In CABI searchRxiv, no results were found.

## Review for urinary tract infection

The terms for the search of this topic in English were: ("cystitis" OR "urinary tract infection" OR "uti" OR "bladder infection" OR "urethra*" OR "ureter*" OR "kidne*" OR "nephr*" OR "renal*" OR "*urinar*" OR "bladder*" OR "vesica urinaria") and terms excluded (NOT) were ("guinea" OR "woman" OR "women" OR "man" OR "men" OR "mice" OR "nursing" OR "patient*" "pigtail" OR "boards" OR "pigmentary"). In a first step, topic in title, animal in keyword and pain in text yielded n=37 results. In a second step, pain was searched in keywords, animal in text and topic in title (exclusion in text) yielding n=118 results. N=10 were selected for screening. Eventually, two papers were selected for the review. In CABI searchRxiv, no papers were found even though terms were searched without field-specification. In German language, topic terms were: *ystitis” OR “harnwegsinfekt*” OR “blasen*” OR “harn*” OR “urethr*” OR “ureter*” OR “Niere*” OR “renal*” or “vesica*” OR “urinaria” OR “Nephr*”. All terms were search in text (n=213) and none paper was selected for screening..In CABI searchRxiv, no results were found with given terms, even without specification.

## Review for mastitis, udder, MMA

The terms for the search of this topic in English were: ("mastitis" OR "MMA" OR "udder" OR "dysgalactiae" OR "agalactiae") and terms excluded (NOT) were ("cow" OR "cattle" OR "dairy"). As many false topics were retrieved, and none was pain-specific, the term pain was deleted, topic and animal were searched keywords and excluded terms in text which n=36 results. One paper was selected for screening and further were retrieved by snowballing. In the additional search in rxiv, n=126 abstracts were found (no specification but only if access is possible) but none was included for the review.

In German language, topic terms were: ("mastitis" OR "MMA" OR "euter" OR "dysgalactiae" OR "agalactiae"). All terms were search in text and one paper was found but not selected. In CABI searchRxiv, five results were found (no specification) but none was selected.
