# Supplementary material for: Part II: understanding pain in pigs—pain assessment in pigs with spontaneously occurring diseases or injuries
Source: Porcine Health Manag. 2025 Mar 12;11:13. doi: 10.1186/s40813-025-00420-1 (PMC11900645; doi:10.1186/s40813-025-00420-1)
Supplement: Supplementary file 2 — Supplementary Material 2 [file 40813_2025_420_MOESM2_ESM.docx]

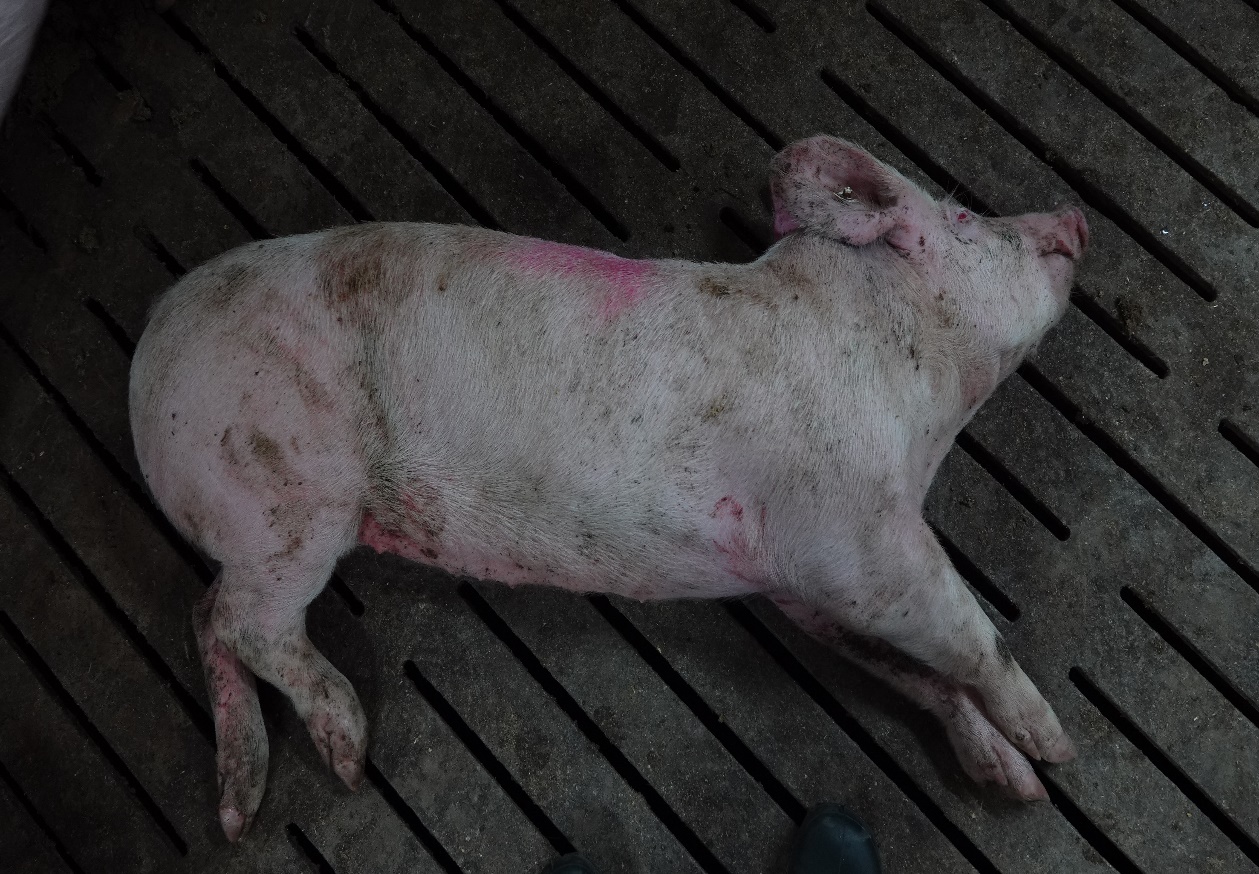


## Additional file 2

**Title name**: **Ulcer of a pig 1**

**Description**: The picture shows a pig with ulcer.


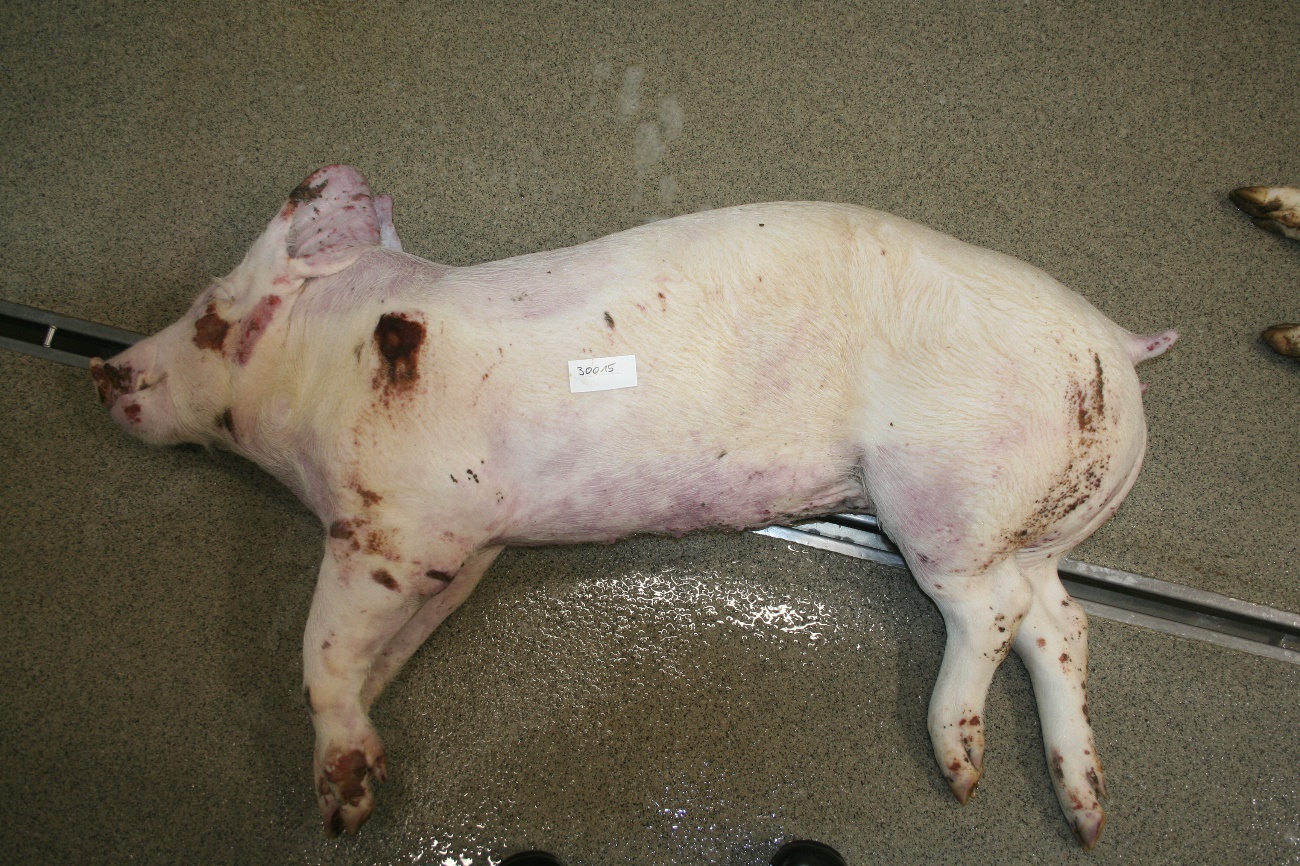


## Additional file 3

**Title name**:  **Ulcer of a pig 2**

**Description**: The picture shows a pig with ulcer.


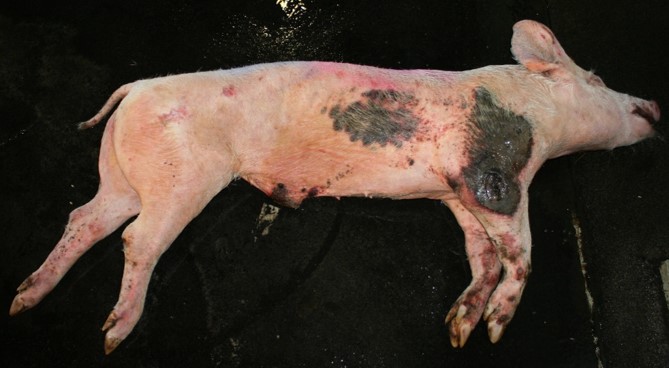


## Additional file 4

**Title name**:  **Ulcer of a pig 3 (figure 3)**

**Description**: The picture shows a pig with ulcer.


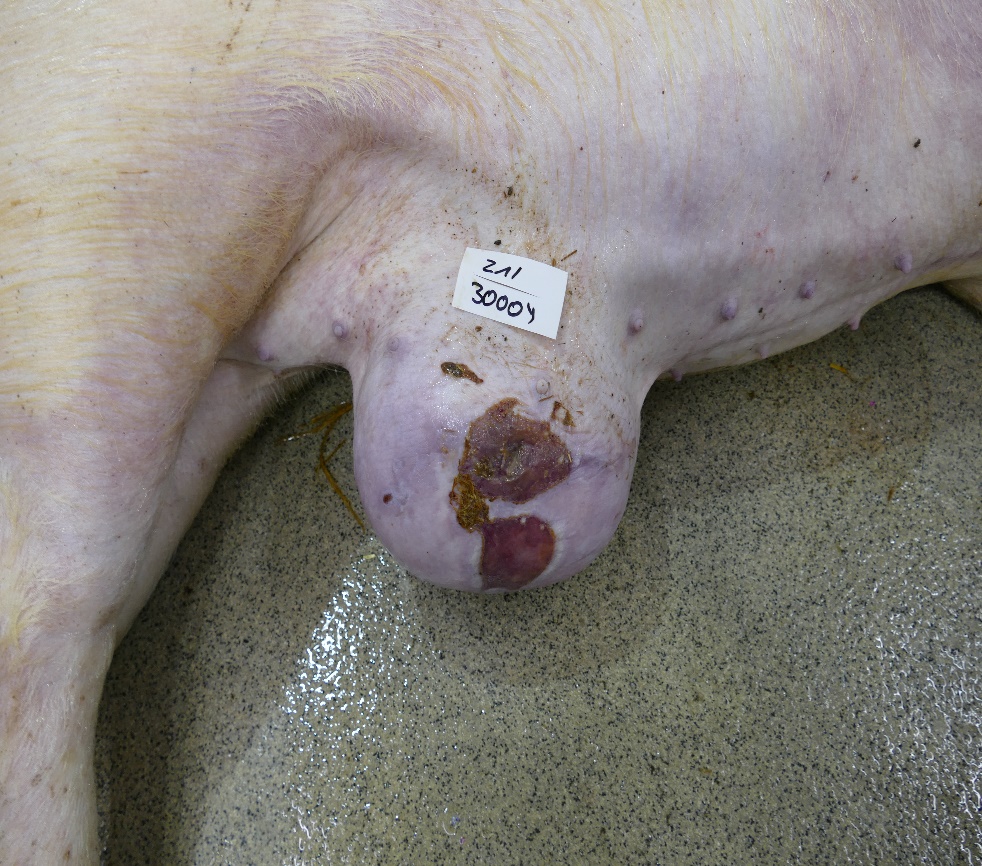


## Additional file 5

**Title name**:  **Umbilical hernia**

**Description**: The picture shows a pig umbilical hernia.
